# Synthesis and Characterization of Graphene Oxide/Chitosan Composite Aerogels with High Mechanical Performance

**DOI:** 10.3390/polym11050777

**Published:** 2019-05-01

**Authors:** Yang Gong, Yingchun Yu, Huixuan Kang, Xiaohong Chen, Hao Liu, Yue Zhang, Yimeng Sun, Huaihe Song

**Affiliations:** State Key Laboratory of Chemical Resource Engineering Beijing University of Chemical Technology, No. 15 North Third Ring Road East, Chaoyang District, Beijing 100029, China; 13126977800@163.com (Y.G.); yuyc@mail.buct.edu.cn (Y.Y.); 13717636585@163.com (H.K.); liuhowe1015@163.com (H.L.); zhangyl918@163.com (Y.Z.); YIMENGSUNNY@163.com (Y.S.); songhh@mail.buct.edu.cn (H.S.)

**Keywords:** chitosan aerogel, graphene oxide, synthesis process, mechanical performance, crystallinity

## Abstract

Chitosan, a semi-crystalline biomolecule, has attracted wide attention due to its high synthesis flexibility. In this study, to improve the mechanical properties of chitosan aerogels (CSAs), graphene oxide (GO) was extracted and introduced into chitosan aerogels as fillers. The porous CSAs/GO composite aerogels were fabricated by an environmentally friendly freeze-drying process with different GO contents (0, 0.5, 1.0, 1.5, wt.%). The characteristics of the CSAs/GO were investigated by scanning electron microscopy (SEM), mechanical measurements and mercury porosimeter. The crystallinity of samples was characterized by X-ray diffraction (XRD). The mechanism of the effect of graphene oxide on chitosan was studied by Fourier transform infrared spectroscopy (FTIR), and X-ray photoelectron spectroscopy (XPS). The results show that the microstructure of the samples is developed in the network structure. The porosity of CSAs/GO aerogels is as high as 87.6%, and the tensile strength of the films increased from 6.60 MPa to 10.56 MPa with the recombination of graphene oxide. The crystallinity (CrI) of composite aerogels increased from 27% to 81%, which indicates that graphene oxide improves the mechanical properties of chitosan by chemical crosslinking.

## 1. Introduction

With the worsening of environmental problems, more and more attention has been attached to green chemistry [1]. Chitosan (CS), a widely used polysaccharide, is the second largest renewable biopolymer after cellulose [2,3]. Chitosan is composed of β-(1-4)-D-glucosamine units and β-(1-4)-N-acetyl-glucosamine depending on the deacetylation of chitin [4]. Contrary to chitin, both amino and hydroxyl groups of chitosan can be selectively modified for versatile applications [5]. The physicochemical characteristics and functional properties of CS such as its biocompatibility, biodegradability, non-toxicity, and film formation ability that are advantageous for industrial and biomedical applications. In recent years, chitosan composite aerogels have attracted wide attention and have been applied in many fields, such as water treatment [6], dye removal [7], tissue engineering [8], and wound healing. Sajjad Keshipour et al. [9] developed a cross-linked chitosan aerogel modified with Au by loading Dimercaprol to use for the catalytic application. Yuqi Li et al. [10] introduced a robust salt-tolerant aerogel for highly efficient oil/seawater separation. P.K. Dutta et al. [11] demonstrated a chitosan-grafted graphene oxide aerogel for adsorption of CO_2_ gas. Jian Feng et al. [12] reported that the addition of polyvinyl alcohol to chitosan aerogels reduces the shrinkage during freeze-drying. Although chitosan has been widely used in many fields, there are few experiments to study its mechanical properties.

Graphene oxide (GO), a two-dimensional honeycomb lattice substance, has attracted significant attention due to its exceptional mechanical, thermal, electronic, electrochemical and optical properties [13,14,15]. In addition, GO also has the characteristics of large surface area, abundant surface chemical groups, strong ion exchange ability and high thermal conductivity, which endow GO/polymer composites with high strength and high modulus, high thermal conductivity and other superior properties. Therefore, GO nanocomposites have become the focus of research in recent years, and more attention has been paid to them [16,17,18]. Stankovich et al. [19] reported that thin layer graphene polystyrene nanocomposites were dispersed in organic solvents, and then graphene polystyrene nanocomposites were prepared. Ke Zhan et al. [20] reported graphene oxide/Al composites with enhanced mechanical properties fabricated by simple electrostatic interaction and powder metallurgy. Ruoff and Aksay et al. [21] demonstrated that the glass transition temperature was raised by adding only graphene into polypropylene and polymethacrylate methyl acetate, and the mechanical and thermal stability properties such as Young’s modulus, tensile strength and thermal stability were improved. Titash Mondal et al. [22] grafted graphene with high density and high anisotropy onto PCL, which led to a conformation change of the polymer chain, improved nucleation process and microcrystalline formation of semicrystalline PCL.

In this paper, a graphene oxide/chitosan composite aerogel (CSAs/GO) with high mechanical properties and porosity was developed by an environmentally friendly freeze-drying process. When graphene oxide was added, the mechanical properties of chitosan aerogels were improved. At the same time, it is worth noting that the crystallinity of chitosan aerogels was also greatly improved. This indicates that graphene oxide not only improves the mechanical properties of chitosan aerogels by physical enhancement, but also improves the mechanical properties of chitosan by chemical enhancement. In addition, we studied the mechanism of the effect of graphene oxide on chitosan by FTIR, XRD and XPS. Under the condition of keeping high porosity, the mechanical properties of chitosan composite aerogels were improved, thus promoting the application of chitosan aerogels in many fields, such as water treatment, catalysis and tissue engineering, and so on. Meanwhile, it was expected that this work could offer a new way of enhancing the properties of chitosan by changing its crystallinity.

## 2. Materials and Methods

### 2.1. Materials

Chitosan was purchased from Sinopharm Chemical Reagent Co., Ltd., Beijing, China, with a deacetylation degree ≥95%. The GO in this work was prepared by the oxidation of graphite powders via a modified Hummers method [23]. Acetic acid was supplied by Sinopharm Chemical Reagent Co. Ltd., Beijing, China. Ethyl alcohol absolute was purchased from Beijing Tongguang Fine Chemical Company, Beijing, China. Deionized and doubly distilled water was employed in all experiments. All reagents in this study were used as received without further purification.

### 2.2. Oxidation of Graphite Powders Process

For oxidation of graphite powders process, the modified Hummers’ method was carried out as follows. Some of the graphite powders, NaNO_3_ powder and concentrated H_2_SO_4_ were added into a 100 mL three-neck flask bottle. The bottle was put into a 0 °C ice water bath and a certain amount of KMnO_4_ was gradually added with stirring. After reaction under 0 °C for 30 min, the temperature was raised to 35 °C and maintained for 96 h. After dilution of the product, addition of excess H_2_O_2_ (30%, *v/v*), and washing to neutral by high speed centrifugation, GO was obtained. The detailed procedure was reported previously by our group [23].

### 2.3. Preparation of CSAs/GO

**Figure 1:**
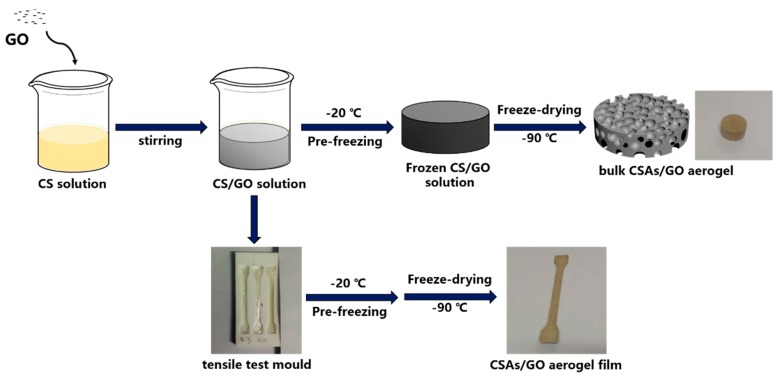
Schematic illustration showing the whole process for the preparation of CSAs/GO.

CSAs/GO was synthesized as follows: GO (0%, 0.5%, 1.0%, 1.5% wt.%) was homogeneously dispersed in 40 mL of deionized water by tip sonication for 1 h, and the samples were denoted as GO-0, GO-0.5, GO-1.0 and GO-1.5, respectively. Then, 1.5 g of chitosan was added to the homogeneous GO solution under vigorous stirring, at room temperature, to obtain a homogeneous CS/GO solution mixture. When GO and CS were dispersed evenly, acetic acid, 0.8 mL, was added with continuous stirring for 12 h. Because of the high synthesis flexibility, we can prepare different shapes of CSAs/GO aerogels through different molds. As shown in Figure 1, we poured the homogeneous CS/GO solution after stirring for 12 h into the ampoule bottles and tensile test molds to generate the bulk CSAs/GO aerogels and CSAs/GO aerogel films, respectively. The resulting CS/GO solution was pre-frozen at −20 °C. The CSAs/GO aerogels were obtained after a freeze-drying process at −90 °C for 48 h. The whole process showing the preparation of CSAs/GO is illustrated in Figure 1.

### 2.4. Characterization

The micromorphology was characterized by a scanning electron microscope (SEM, ZEISS, SUPRA-55, Jena, Germany). The chemical group was investigated by Fourier transform infrared spectroscopy (FTIR, NICOLET-iS50, Madison, Wisconsin, America). The pore structure of the samples was characterized by a mercury porosimeter (AutoPore Iv 9510, Atlanta, Georgia, America). X-ray photoelectron spectroscopy (XPS) analysis was conducted on a Thermo Scientific Escalab 250Xi (Boston, Massachusetts, America) equipped with a monochromatic Al-Kα X-ray source. XPSPEAK4.1 was applied to fit the XPS results.

### 2.5. Mechanical Test

Mechanical tests of CSAs/GO aerogels were performed on a universal testing machine at room temperature (5567, Instron, Boston, Massachusetts, America). The samples for mechanical test were prepared following the illustration in Section 2.3. We used bulk CSAs/GO aerogels for compression tests and CSAs/GO aerogel films for tensile tests. As shown in Figure 1, the bulk CSAs/GO aerogel is a cylinder with a diameter of 20 mm and a height of 7 mm, and the thickness of CSAs/GO aerogels films is 100–200 μm and the width is 5 mm. The compression test rate is 10 mm/min, and the tensile test rate is 20 mm/min.

### 2.6. Testing of Crystallinity

Crystallinity is the mass fraction or volume fraction of crystalline regions in the polymer [24].

X_C_ = W_c_/W × 100% (1)

W is the total mass of the polymer and W_c_ is the mass of crystalline part of the polymer sample.

X-ray diffraction (XRD) is the most direct method to study the crystal structure of chitosan, and it is also the most effective method to calculate the crystallinity of chitosan. The principle is to calculate the crystal cell and crystallinity parameters in the molecular chain according to the intensity and position of the strongest diffraction peak of chitosan. According to reference [25], the crystallinity of chitosan can be calculated by Formula (2).

(2) $$\mathrm{CrI}\left(\%\right)=\frac{\left(I_{110}-I_{am}\right)}{I_{110}}\times 100\%$$

*I*_110_ represents the maximum absorption intensity during 19°–20° and *I*_am_ represents 12.6° absorption intensity.

In this work, testing of crystallinity of CSAs/GO was performed by X-ray diffraction (XRD, D/max-2500B2t/PCX, Tokyo, Japan).

## 3. Results and Discussion

**Figure 2:**
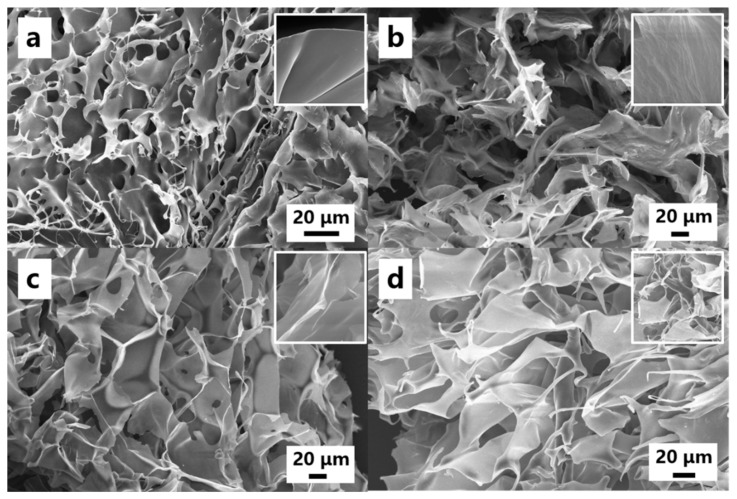
SEM images of the CSAs/GO aerogels. (**a**) GO-0, (**b**) GO-0.5, (**c**) GO-1.0, (**d**) GO-1.5, and the insets are selected magnifications.

The microstructure of the CSAs/GO aerogels with different GO contents is shown in Figure 2. It can be clearly seen that the aerogel has a well-connected 3D porous structure. Numerous macropores in the size range 10–50 μm are randomly distributed in the CSAs aerogel (Figure 2a). After the addition of GO, The CSAs/GO aerogel also displays a highly porous and interconnected structure; at the same time, it can be seen that with the addition of GO, the microstructure of the samples gradually changes to lamellar (Figure 2b–d). The images inside are magnifications of different samples; it can be seen that with the addition of GO, the surface of the samples becomes rougher. The above phenomenon shows that the addition of GO has an effect on the micromorphology of chitosan.

**Figure 3:**
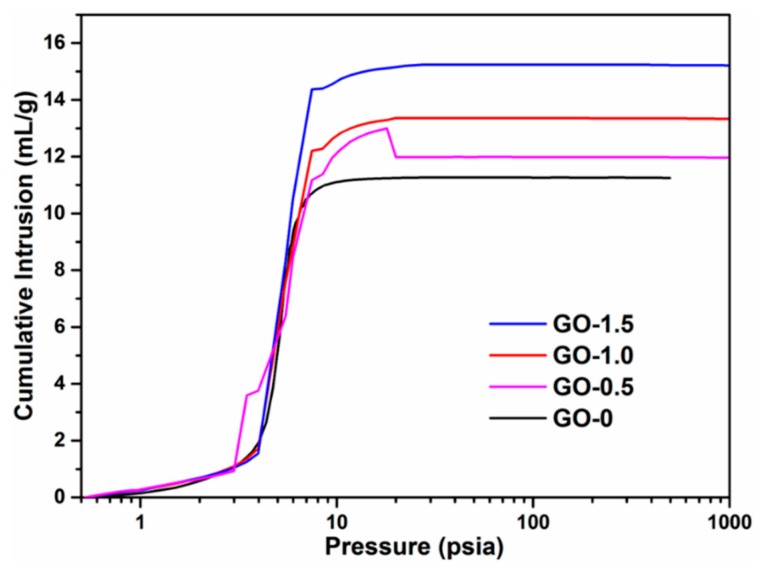
The mercury porosimeter curves of CSAs/GO aerogels.

The porosity of the CSAs/GO aerogels was determined by a mercury porosimeter. As shown in Figure 3, with the addition of GO, the cumulative intrusion of the sample increases gradually, which indicates that the porosity of the sample is increasing. The results of the mercury porosimeter are displayed in Table 1. From Table 1, the total pore volume and total pore area of the samples are increasing. The porosities of samples are 83.7%, 85.2%, 87.2%, and the porosity of GO-1.5 is as high as 87.6%, which also demonstrates that the CSAs/GO aerogels have a well-connected 3D porous structure, as shown in SEM images.

**Figure 4:**
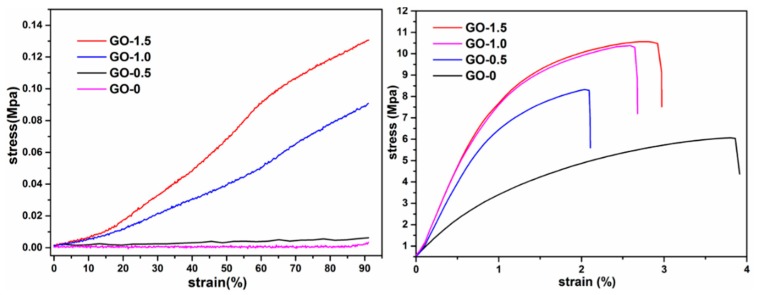
The compressive stress-strain curves (**left**) and tensile stress-strain curves (**right**) of CSAs/GO aerogels.

**Figure 5:**
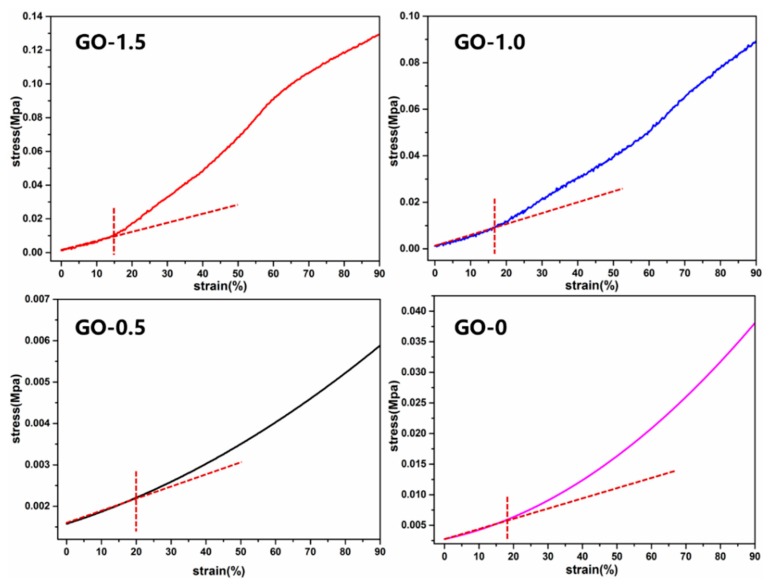
The elastic region for CSAs/GO aerogels.

The mechanical performance of the CSAs/GO aerogel was also important. Figure 4 shows the compressive stress-strain curves (left) and tensile stress-strain curves (right) of CSAs/GO aerogels. The compressive stress-strain curves were measured by a dynamic mechanical analyzer with 90% compression strain. As is shown in Figure 4 (left), with the addition of graphene oxide, the stress required to produce the same strain increases gradually. Compared with GO-0 and GO-0.5, the compression resistance of GO-1.0 and GO-1.5 has been greatly improved, mainly because graphene oxide plays a supporting role in the network skeleton of chitosan. Figure 5 shows the elastic region for each sample; it can be seen that the linear elasticity feature is revealed at low strains of 15%–20% for each sample. This is attributable to elastic deformation at an early strain stage. It can also be seen that the elastic range of GO-0 and GO-0.5 is larger than that of GO-1.0 and GO-1.5, which is also caused by the addition of graphene oxide. As the compression of the CSAs/GO aerogel increases, it can be seen from Figure 4 (right) that the tensile strength of the samples has also been greatly improved. The tensile strength increased from 6.06 MPa of GO-0 to 10.56 MPa. of GO-1.5, as show in Table 2. It can also be seen that the tensile strain of the sample decreases with the addition of graphene oxide, which indicates that the sample becomes stiffer after adding GO, which also confirms the conclusion from the compressive stress-strain curves. The latter analysis demonstrates that the carboxyl group of graphene oxide reacts with the amino group of chitosan, which changes the crystallinity of chitosan and also plays the role of crosslinking. The uniformly dispersed graphene oxide was embedded in the structure of chitosan, so its tensile strength was greatly improved. Combined with the results of Mercury instruments, CSAs/GO aerogels not only improve the porosity, but also the mechanical properties of aerogels, which has great significance for the application of aerogels in the fields of adsorption, catalysis and so on.

**Figure 6:**
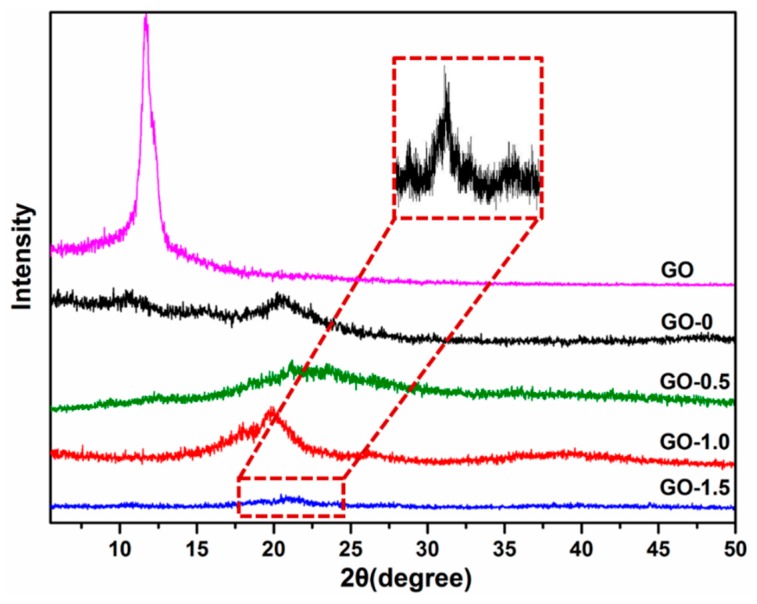
XRD patterns of GO and CSAs/GO aerogels with different GO contents.

It can be seen that the mechanical properties of the CSAs/GO aerogels increased obviously after the addition of GO. In addition to physical enhancement of GO, GO also improves the mechanical properties of aerogels through chemical forms. We found that with the addition of GO, the crystallinity of chitosan aerogels was greatly improved. XRD was used to further investigate the crystallinity of the as-synthesized CSAs/GO aerogels. As shown in Figure 6, GO exhibited one obvious XRD diffraction peak centered at 2θ = 10° assigned to (100) lattice planes [26]. However, there was no sharp peak at 2θ = 10° of CSAs/GO aerogels, which indicated that GO formed a good compound with chitosan in the interior of the chitosan. CS has a wider diffraction peak at 2θ = 21.5°, which is a characteristic peak of chitosan. The crystallinity of each sample is calculated by formula 2, and the results are shown in Table 3. It can be seen that the results of crystallinity indicate the change of the XRD pattern. After adding graphene oxide, the crystallinity of the sample increases from 23.7% to 59.5%, and the crystallinity of GO-1.5 increases obviously, while the crystallinity of GO-1.5 increases to 81.1%. The crystallinity of chitosan is determined by the intramolecular and intermolecular hydrogen bonds of its tertiary structure [27]. Researchers found that the crystallization of chitosan was improved by the treatment of hydrogen bond breaking compounds such as ammonia and sodium hydroxide [28]. In this experiment, GO not only affected the crystallization of chitosan as a nucleating agent [22] during the process of freeze-drying, but also affected the formation of hydrogen bonds of chitosan by reaction with the amino group. The change of hydrogen bonds also led to the change of the crystallinity of chitosan.

**Figure 7:**
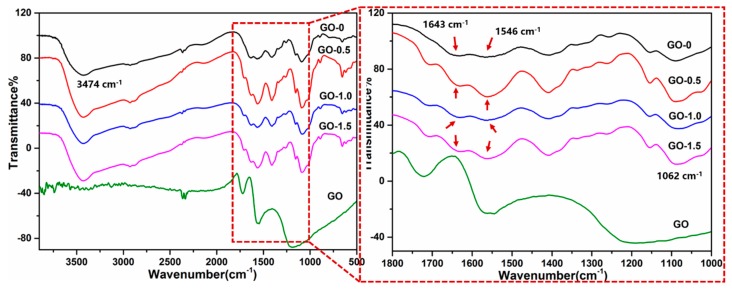
The FTIR spectrum of CSAs/GO aerogels.

FTIR and XPS were utilized to further explore the interaction between graphene oxide and chitosan. Figure 7 demonstrates the FTIR spectrum of different groups of the CSAs/GO aerogels and the spectrum on the right is the detailed figure which focused in the spectral range (~1800 to 1000 cm^−1^). As shown in the FTIR spectrum, the band monitored at 3474 cm^−1^ is assigned to the stretching vibrations for N-H and O-H and the peak at 1062 cm^−1^ corresponds to C-O stretching vibration peaks over secondary alcohols [29]. The absorption band at 1643 cm^−1^ is characteristic of the carbonyl stretching mode of amide groups; and the other at 1546 cm^−1^ is related to the bending mode of the primary amine and to the protonated amine [21]. In both cases, the band at 1643 cm^−1^ is related to acetylated units, but for CSAs/GO aerogels it is also related to the amide group formed by the reaction of chitosan with carboxyl of GO [30], as shown in Figure 7. The reaction of chitosan with GO is confirmed by an increase in the ratio calculated by the Formula (3) [31] and the values of *A*_1643_ and *A*_1546_ for each sample are shown in Table 4. The calculated results are shown in Table 5. It can be seen that the values of R increase from 1.27% to 7.98%, which indicates the increase of amide groups in CSAs/GO aerogels and also confirms the reaction between the amino group of chitosan and the carboxyl group of GO.

(3) $$R=\frac{A_{1643}-A_{1546}}{A_{1546}}$$

where *A*_1643_ and *A*_1546_ are absorption peaks of amide groups (1643) and primary amine (1546).

**Figure 8:**
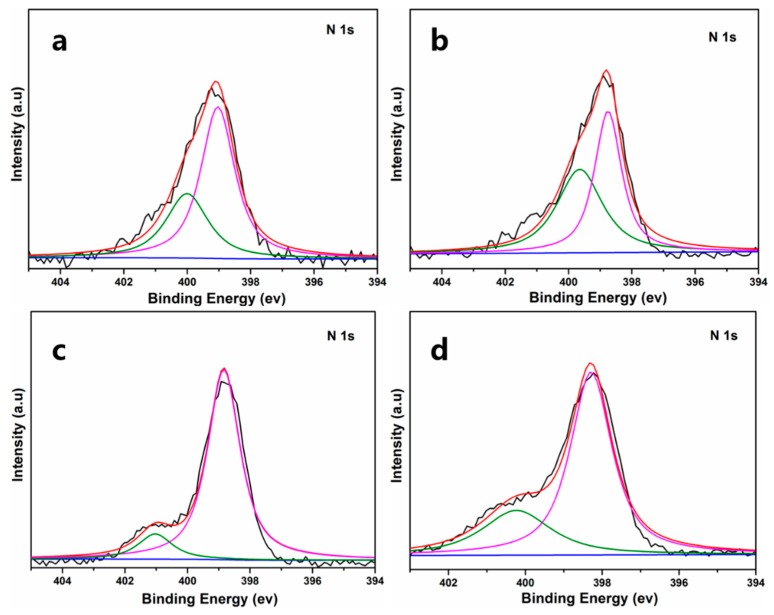
XPS spectra of the N 1s region for (**a**) GO-0, (**b**) GO-0.5, (**c**) GO-1.0 and (**d**) GO-1.5.

Figure 8 shows the XPS results of CSAs/GO aerogels. There are two convoluted peaks in the N 1s spectra for GO-0, GO-0.5, GO-1.0 and GO-1.5. The peak at 399.5 eV is related to amine (primary and tertiary) and amide groups, and the other, at 400.8 eV, is related to protonated amine (-NH_3_^+^) [32]. If the amino group of graphene oxide reacts with the carboxyl group of chitosan, the area ratios of A399.5 and A400.8 will increase gradually because the amount of amide groups will increase. The results of the ratio are shown in Table 6. It can be seen that the ratio increases with the addition of graphene oxide and the results are consistent with the results of FTIR analysis, which further confirms the reaction between the amino group of chitosan and the carboxyl group of GO.

**Figure 9:**
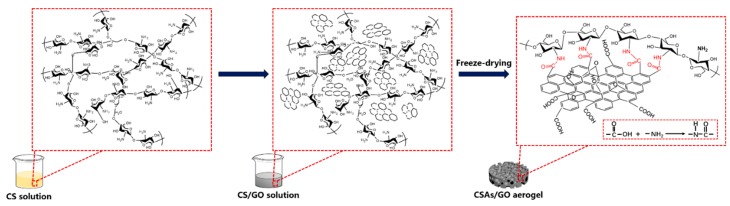
Mechanism diagram of reaction between GO and chitosan.

We can see from the results of XRD, FTIR and XPS that when graphene oxide is combined with chitosan, the carboxyl group on GO reacts with the amino group in chitosan, as shown in Figure 9. In CS solution, the molecular chains of chitosan were disordered, showing an amorphous state, while in CS/GO solution, graphene oxide was uniformly dispersed among chitosan molecules. In the freeze-drying process, chitosan molecules preferentially precipitated at graphene oxide, which can be nucleated to a certain extent. At the same time, the amino groups on chitosan reacted with the carboxyl group of graphene oxide, which made chitosan molecules distribute regularly on the surface of graphene oxide; the formation of hydrogen bonds in the freeze-drying process will be affected because of this reaction, so the crystallinity of composite aerogels has been greatly improved. Therefore, the improvement of the mechanical properties of CSAs/GO aerogels is due to the interaction with graphene oxide. The interaction with graphene oxide also increases the well-connected 3D porous structure and porosity of the CSAs/GO aerogels, which has great significance for the application of aerogels in the fields of adsorption, catalysis and so on.

## 4. Conclusions

Graphene oxide/chitosan composite aerogels with high mechanical properties and porosity were successfully prepared by an environmentally friendly freeze-drying process. The microscopic analysis shows that the prepared samples have a well-connected 3D porous structure, and the porosity of the samples increases from 83.7% to 87.6% with the addition of graphene oxide. The mechanical properties demonstrate that the compressive strength of the samples increases with recombination of graphene oxide, and the tensile strength of the films increased from 6.60 MPa to 10.56 MPa. The results of XRD, FTIR and XPS show that when graphene oxide is combined with chitosan, the carboxyl group on GO reacts with the amino group in chitosan, which indicates that graphene oxide not only improves the mechanical properties of chitosan aerogels by physical enhancement, but also improves the mechanical properties of chitosan by interaction with GO. Under the condition of keeping high porosity, the mechanical properties of chitosan composite aerogels were improved, thus promoting the application of chitosan aerogels in many fields.

## Figures and Tables

**Table 1:** The results of the mercury porosimeter.

Samples	Total Pore Volume (mL/g)	Total Pore Area (m^2^/g)	Porosity (%)
GO-0	11.271	1.264	83.7
GO-0.5	12.995	1.591	85.2
GO-1.0	13.243	1.625	87.2
GO-1.5	15.246	1.858	87.6

**Table 2:** The tensile strength results.

Samples	GO-0	GO-0.5	GO-1.0	GO-1.5
maximum load (MPa)	6.06	8.32	10.34	10.56

**Table 3:** The crystallinity of CSAs/GO aerogels with different GO contents.

Samples	GO-0	GO-0.5	GO-1.0	GO-1.5
*CrI* (%)	23.7	59.5	72.5	81.1

**Table 4:** The values of *A*_1643_ and *A*_1546_ for each sample.

Samples	GO-0	GO-0.5	GO-1.0	GO-1.5
*A*_1643_	71.711	52.088	78.543	71.691
*A*_1546_	70.812	51.047	76.478	66.696

**Table 5:** The value of R of CSAs/GO aerogels with different GO contents.

Samples	GO-0	GO-0.5	GO-1.0	GO-1.5
*R* (%)	1.27	2.04	2.70	7.98

**Table 6:** The area ratio of A399.5 and A400.8.

Samples	GO-0	GO-0.5	GO-1.0	GO-1.5
*R*	0.98	1.91	2.48	7.22
